# Integrated Enzyme-Mediated One-Step Sample Processing and Duplex Amplification System for Rapid Detection of *Carpione rhabdovirus* in Aquaculture-Derived Food Products

**DOI:** 10.3390/foods14223929

**Published:** 2025-11-17

**Authors:** Heng Sun, Haoyu Wang, Jie Huang, Yao Wu, Zhenxin Hu, Yucong Huang

**Affiliations:** 1 Guangdong Provincial Key Laboratory of Aquatic Animal Disease Control and Healthy Culture & Key Laboratory of Control for Disease of Aquatic Animals of Guangdong Higher Education Institutes, Fisheries College of Guangdong Ocean University, Zhanjiang 524088, China; hengsuen@foxmail.com (H.S.); haoyuwangmail@foxmail.com (H.W.);; 2 GeneVide Biotech Co., Ltd., Suzhou 210500, China

**Keywords:** one tube rapid detection, enzyme-mediated one-step sample processing, enzymes-mediated duplex exponential amplification

## Abstract

Golden pompano (*Trachinotus ovatus*) is the largest-scale marine aquaculture fish species in China, with a significant economic and nutritional value as a high-quality seafood product. The recent outbreak of an epidemic caused by a novel *Carpione rhabdovirus* (CAPRV) occurred in cultured golden pompano. To address it, a CAPRV enzyme-mediated one-step sample processing–reverse transcription–enzyme-mediated duplex exponential amplification (EmOSP-RT-EmDEA) detection system was developed. This innovative molecular diagnostic tool integrates enzyme-mediated one-step sample processing (EmOSP) with enzyme-mediated duplex exponential amplification (EmDEA) technology. Unlike traditional RPA-Cas12a detection methods, this system directly incorporates fluorophores into RNA components, eliminating the need for exogenous fluorescent probes while maintaining high sensitivity. It enables rapid, sensitive, and specific detection of CAPRV2023 across various sample types, including clinical, invasive, minimally invasive, and environmental specimens. Performance evaluation of the CAPRV2023 EmOSP-RT-EmDEA detection system against conventional diagnostic methods, such as TaqMan qPCR and traditional PCR, demonstrated superior sensitivity, with a detection limit as low as 4 copies/μL, and exceptional specificity. The optimized EmOSP protocol for nucleic acid extraction from fecal, hepatic, and water samples provided robust and reproducible results. The EmOSP-RT-EmDEA system achieved a detection rate of 68.14% in fecal samples, matching the performance of the gold-standard TaqMan qPCR assay.

## 1. Introduction

*Trachinotus ovatus*, commonly known as golden pompano, is a species in the genus *Trachinotus* of the Carangidae family, widely distributed in subtropical and tropical seawaters of Southeast Asia. In recent years, due to its rapid growth, strong adaptability, and high economic value, golden pompano has become the most widely cultivated fish species in net cage aquaculture along China’s southern coast. According to data from the China Fisheries Statistical Yearbook 2024, the cultured production of golden pompano in China reached 292,263 tons in 2023 [1,2,3,4].

In the autumn of 2023, a novel acute infectious disease emerged among cage-farmed golden pompano in southern China, resulting in mortality rates ranging from 65% to 82% within just 7 to 10 days. This outbreak posed a significant threat to China’s marine aquaculture industry [1,2]. A novel rhabdovirus strain was isolated from the infected golden pompano and designated as *Carpione rhabdovirus* 2023 (CAPRV2023) [1,2]. CAPRV virions are enveloped, bullet-shaped particles (approximately 183–218 × 57–83 nm) containing a negative-sense single-stranded RNA genome (~11.7 kb) with the canonical 3′-N-P-M-G-NV-L-5′ gene organization, characteristic of the genus *Novirhabdovirus*. CAPRV2023 represents only the second recorded strain of this viral species, following the initial isolation of strain CAPRV583 in Italy from the carpione (*Salmo trutta carpio*) [1,2,3]. Notably, the whole-genome similarity between CAPRV2023 and CAPRV583 is only 81.32% [1,2,3].

Due to the lack of antiviral drugs and corresponding vaccines, there are currently no effective control measures for this emerging infectious disease. Therefore, there is an urgent need to develop precise and sensitive molecular assays to detect CAPRV2023 as early and accurately as possible. Early detection is crucial for enabling effective prevention and control measures. To date, in addition to traditional necropsy and pathological examination, Sun et al. have developed various diagnostic methods for detecting CAPRV2023, including cell culture, in situ hybridization, electron microscopy, reverse transcription polymerase chain reaction (RT-PCR) and SYBR Green I quantitative PCR (qPCR) [1,2,3].

Recombinase polymerase amplification (RPA) is an emerging isothermal nucleic acid amplification method for pathogen detection that utilizes recombinase, single-strand binding protein (SSB), and DNA polymerase [5,6]. Combined with fluorescent probes or lateral flow strips, RPA enables rapid on-site detection of pathogens such as SARS-CoV-2 and Zika virus. However, its high sensitivity can sometimes lead to nonspecific amplification and compromise accuracy [5,6,7].

The CRISPR-Cas12 system, known for its high sensitivity and specificity, is a promising tool for genome editing, diagnostics, and pathogen detection [8,9]. RPA rapidly amplifies nucleic acids at constant temperatures, and CRISPR effectors (e.g., Cas12 or Cas13) confirm amplified products, amplify the signal, and reduce false positives. The combination of RPA and CRISPR enables faster, simpler, and more efficient assays, improving the specificity and sensitivity of nucleic acid detection in field and resource-limited settings, such as disease screening, food safety, and environmental monitoring [8,9,10]. However, maintaining RPA enzyme activity requires long-term storage at −20 °C, posing challenges for practical applications. Additionally, the RPA-CRISPR-Cas12a system involves multiple steps (nucleic acid extraction, RPA, and Cas12a binding), each requiring tube opening, which increases the risk of aerosol contamination and lengthens testing time [11,12].

To ensure accurate detection of aquatic pathogens, complex sample processing is required to obtain high-purity and high-concentration nucleic acids. Typical nucleic acid extraction methods for aquatic samples include centrifuge column-based [13] and magnetic bead-based [14] approaches. Some studies have also reported using direct thermal lysis to release nucleic acids, aiming to simplify sample processing [13]. However, these detection methods are intricate, time-consuming, and generally require well-trained personnel in specialized laboratories. Therefore, it is crucial to develop a new, simple, and rapid detection method for field detection of CAPRV2023, after RNA extraction, allows subsequent reverse transcription and amplification to be performed within a closed, one-step reaction system to meet urgent clinical diagnostic needs.

**Figure 1:**
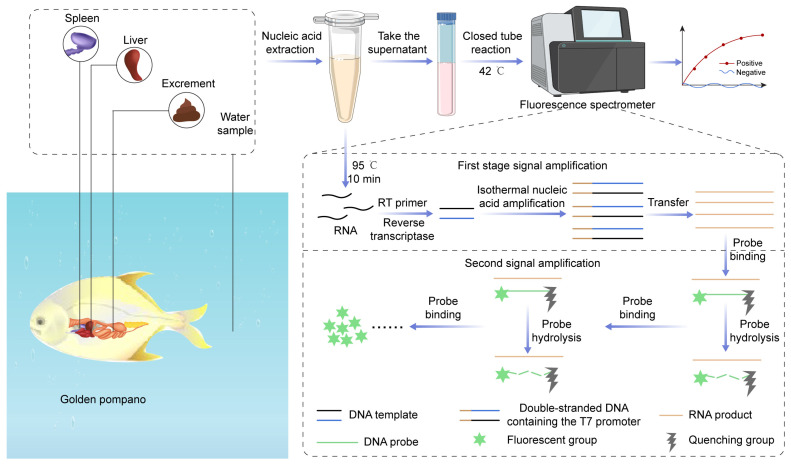
**Operating procedure and technical principles of the CAPRV2023-EmOSP-RT-EmDEA detection system.** Initially, RNA is reverse-transcribed into complementary DNA (cDNA) using reverse transcriptase in the presence of RT primers. Simultaneously, EmDEA primers bind to the target cDNA with recombinase to search for homologous sequences. Upon recognition of the homologous sequence, the recombinase invades the DNA duplex to form a D-loop structure, allowing the primer to be extended by SAP polymerase to generate new strands. This process is repeated to complete DNA replication. During amplification, one of the primers contains a T7 promoter. The amplification product bearing T7 promoter is efficiently transcribed into RNA by T7 RNA polymerase, yielding single-stranded RNA. The RNA probe binds to the newly generated RNA strand and is cleaved explicitly by the signal amplification enzyme. This cleavage separates the fluorescent group from the quencher group, producing a fluorescent signal. After cleavage, the stability of the bound RNA probe decreases, facilitating the rebinding of the RNA probe to the RNA strand in solution. The signal amplification enzyme cleaves the RNA probe again, generating additional fluorescence signals.

The present study introduces an innovative method based on a dual-signal amplification technique for nucleic acid detection of CAPRV2023 within a closed-tube system. The newly developed enzyme-mediated one-step sample processing–reverse transcription–enzyme-mediated duplex exponential amplification (EmOSP-RT-EmDEA) detection system integrates enzyme-mediated lysis and isothermal duplex exponential amplification into a single, closed-tube reaction. This system enables rapid, RNA extraction-free detection via a simplified one-step process. A concise overview of the reaction mechanism, including the role of RNA primers and the signal amplification strategy, is presented in Figure 1. This repetitive cycle effectively amplifies the detection signal, as illustrated in Figure 1.

This study presents an integrated CAPRV2023 EmOSP-RT-EmDEA detection method designed to streamline and enhance CAPRV2023 detection. The procedure begins with rapid RNA release from clinical samples of golden pompano using enzyme-mediated one-step sample processing (EmOSP) reagents. The resulting supernatant is directly introduced into the CAPRV2023 RT-EmDEA reaction, performed in a single closed-tube system at a constant temperature of 42 °C. Accurate results are obtained within 30 min, offering a powerful tool for monitoring and controlling CAPRV2023.

## 2. Materials and Methods

### 2.1. Clinical Sample Collection and RNA Preparation

Golden pompano exhibiting clinical symptoms of CAPRV2023 infection were collected from 37 geographically dispersed offshore cage farming sites across China between August 2023 and January 2025. A total of 113 clinical samples were obtained, with approximately three fish sampled per location. To minimize sampling bias, fish were randomly selected from each site regardless of size or sex. This sampling strategy was specifically designed to represent the natural variation in field conditions. The sampling period covered more than one complete production cycle of golden pompano, and the sampled fish represented all developmental stages, providing a comprehensive dataset across time, space, and developmental phases. RNA extraction was conducted following the protocol described by Sun et al. [1,2]. The CAPRV2023 strain was isolated from the affected golden pompano and preserved in the laboratory in 2023 [1,2].

### 2.2. Construction of Plasmid for Standard Quantification

A standard plasmid was constructed using CAPRV-740F/R primers (Table 1) following the protocol described by Sun et al. [1,2]. The plasmid concentration was measured at 136 ng/μL, corresponding to approximately 4.98 × 10^10^ copies/μL. It was then diluted to an appropriate concentration for use in subsequent experiments.

### 2.3. EmDEA Reaction System Development

#### 2.3.1. Design of EmDEA Primers

Six forward primers, six reverse primers, and six RNA primers were designed based on the G protein gene sequence of CAPRV2023 (GenBank accession no. PP050495.1). The sequences are listed in Table 1.

#### 2.3.2. Screening of EmDEA Reaction and Primers

The EmDEA reaction and primer screening were performed using the EmDEA Fluorescent Isothermal Amplification Screening Kit (GeneVide Biotech Co., Ltd., Suzhou, China), which contains enzyme dry powder and an activation solution. The components of the EmDEA Fluorescent Isothermal Amplification Screening Kit include: DNA amplification enzyme system (DNA recombinase, single-strand binding protein, T7 RNA polymerase, ATP regenerating enzyme, reverse transcriptase); signal amplification enzyme system (locating enzyme, cleavage enzyme, shrimp alkaline phosphatase, duplex-specific nuclease); and activation solution (nucleoside triphosphates, NTPs).

Each reaction mixture had a total volume of 20 μL, consisting of 1 μL of forward and reverse primers (10 μM each), 1 μL of RNA primer (100 μM), 7 μL of template DNA, enzyme dry powder, and 10 μL of activation solution.

A plasmid template containing 10^4^ copies was used for RNA probes screening, while decreasing concentrations of 10^3^ and 10^2^ copies were applied for forward and reverse primer screening, respectively. The reaction mixtures were incubated in a LightCycler^®^ 96 real-time quantitative PCR system (Roche, Basel, Switzerland) at a constant temperature of 42 °C, with fluorescence signals recorded every 60 s. The minimum threshold time (Tt) and maximum fluorescence intensity obtained were chosen as screening criteria. Primer combinations that performed well were further tested using a plasmid with only 10 copies, and only those demonstrating reliable amplification at this low copy number were selected for subsequent experiments.

### 2.4. Development of RT-EmDEA Reaction System

#### 2.4.1. Preparation of RNA Standard Samples

RNA from tissues of CAPRV2023-infected golden pompano was quantified using a qPCR method developed by Sun et al. [1,2]. The Ct value was 15.74, corresponding to 10^7.11^ copies. This sample was diluted to the appropriate concentration for further use.

#### 2.4.2. Reverse Transcription Primer Design and RT-EmDEA Reaction

To enable reverse transcription and the EmDEA reaction in a single closed tube, 20 short-sequence reverse transcription primers (Table 2) were designed and synthesized by Sangon Biotech Co., Ltd. (Shanghai, China) and prepared as 400 μM solutions. These primers were mixed equally to create an RT primer mix at a final concentration of 20 μM.

The RT-EmDEA reaction differs from the standard EmDEA reaction by incorporating reverse transcriptase into the enzyme dry powder. The final RT-EmDEA reaction mixture contained 1 μL of the RT primer mix, 1 μL each of forward and reverse primers (10 μM), 1 μL of RNA primer (100 μM), 6 μL of RNA template, and 10 μL of activation solution. The reaction mixture was incubated at 42 °C in a LightCycler^®^ 96 real-time qPCR system (Roche), with fluorescence readings collected every 60 s.

#### 2.4.3. Screening of Compatible Primer Pairs for RT-EmDEA of CAPRV2023

The standard RNA sample prepared in Section 2.4.1 was diluted to 10^5^ copies as a template. Primer combinations selected in Section 2.3.2 were tested in the RT-EmDEA reaction following the methods described in Section 2.4.2 to assess compatibility. An effective primer pair generated a significant amplification curve, and the primer combination with the lowest Tt value was retained for subsequent steps.

#### 2.4.4. Sensitivity of the CAPRV2023 RT-EmDEA Reaction

The standard RNA sample prepared in Section 2.4.1 was serially diluted to 5, 10, 10^2^, 10^3^, 10^4^, and 10^5^ copies and used as a template in the RT-EmDEA reaction to determine the system’s sensitivity.

### 2.5. Enzyme-Mediated One-Step Sample Processing Technique

#### 2.5.1. Sample Processing Method

Three sample processing methods (Methods 1, 2, and 3) were developed to accommodate various experimental requirements. All methods utilized 1 mL of EmOSP lysis solution prepared in 1.5 mL Eppendorf tubes containing consistent components: 20 mM Tris-HCl (pH 8), 300 mM NaCl, 1 mM EDTA, 1% SDS, 0.8% PVP-40, and one engineered proteinase. The only difference among the three methods was the type of enzyme used: Method 1 used thermostable pronase, Method 2 used high-efficiency proteinase K, and Method 3 used thermostable papain. All reagents, including the enzymes, were purchased from GeneVide Biotech Co., Ltd., Suzhou, China. Samples were heated at 95 °C for 10 min, then cooled to room temperature, and the supernatant was collected for detection assays.

#### 2.5.2. Minimally Invasive Sample Extraction

A 10 mg sample of gill filaments and feces from infected fish were collected and processed using each of the three methods described in Section 2.5.1. A 6 μL aliquot of the supernatant was then used in the RT-EmDEA reaction (described in Section 2.4.2) to assess the effectiveness of EmOSP. Each experiment was performed in triplicate. To ensure the reliability of sample extraction, the sample was also quantified according to the method described by Sun et al. [1,2].

#### 2.5.3. Invasive Sample Extraction

Due to the high concentrations of CAPRV2023 in the spleen and liver, 10 mg of tissue samples from both tissues were collected and processed using the three methods described in Section 2.5.1. A 6 μL aliquot of the supernatant was analyzed using the RT-EmDEA reaction as outlined in Section 2.4.2. Each experiment was performed in triplicate, and quantification was confirmed following the method described by Sun et al. [1,2].

#### 2.5.4. Environmental Sample Extraction

For environmental samples, 100 mL of seawater was collected from aquaculture cages where golden pompano were present and first filtered through a 0.22 μm pore-size membrane (Millipore, Burlington, MA, USA) to remove debris and particulates. The filtrate was then divided into two portions: one was directly used for EmOSP-based detection as described in Section 2.5.1, while the other was subjected to viral concentration using a 10 kDa molecular weight cut-off ultrafiltration tube (Millipore). The concentrated viral suspension was subsequently used for RNA extraction and RT-qPCR analysis. A 6 μL aliquot of the resulting supernatant was used for the RT-EmDEA reaction [1,2].

#### 2.5.5. Optimization of EmOSP for Environmental Samples

Given that seawater salinity can influence EmOSP, the salinity of seawater was measured, and sterile seawater with matching salinity was prepared to dilute the test samples. CAPRV2023-containing seawater samples were diluted to various volumes and processed as described in Section 2.5.1. Optimization was assessed based on Tt and fluorescence values, with each test conducted in triplicate. Quantification was performed following the method outlined by Sun et al. [1,2].

### 2.6. EmOSP-RT-EmDEA Detection System Development for CAPRV2023

#### 2.6.1. Integrating RT-EmDEA System for CAPRV2023

The EmDEA primer combination F2-R2-RNA4, along with the 20 RT primers and all RT-EmDEA enzyme components, were formulated into a single dry powder tube by GeneVide Biotech Co., Ltd. (China). For detection, only the test sample and activation solution are needed to initiate the reaction.

#### 2.6.2. Specificity of the EmOSP-RT-EmDEA Detection System for CAPRV2023

RNA was extracted from viral pathogens, including VHSV, SCRV, SVCV, IHNV, NNV, and TiLV, and reverse-transcribed to cDNA. Additionally, DNA from ISKNV, SGIV, bacterial DNA from *Streptococcus dysgalactiae*, *Vibrio alginolyticus*, *Streptococcus agalactiae*, *Streptococcus iniae*, *Lactococcus garvieae*, *Vibrio harveyi*, and golden pompano DNA were tested. The cDNA from CAPRV2023-infected pompano was used as the positive control. Healthy fish cDNA and distilled water served as negative controls. Each experiment was performed in triplicate.

#### 2.6.3. Repeatability of the EmOSP-RT-EmDEA Detection System for CAPRV2023

The system’s repeatability was assessed through inter- and intra-assay tests using the same sample. These tests were performed in triplicate across 10 independent runs in accordance with the protocols outlined in Section 2.5.3 and Section 2.4.3. The coefficient of variation (CV%) was calculated based on the mean Ct values.

#### 2.6.4. Application of the CAPRV2023 EmOSP-RT-EmDEA Detection System

A total of 113 samples, including tissues, feces, and aquaculture water, were randomly collected from golden pompano aquaculture systems in Guangdong and Guangxi provinces between August 2023 and January 2025 for epidemiological surveillance and method comparison. These samples were tested using the CAPRV2023 EmOSP-RT-EmDEA Detection System, TaqMan qPCR, and conventional PCR methods [1,2]. Diagnostic sensitivity and specificity were determined as per OIE guidelines [14]. Viral infection was confirmed by observing typical cytopathic effects (CPEs) in FHM cells inoculated with the samples, which were used to verify CAPRV2023 infection status [1,2]. A Chi-square test was used to compare the detection rates of conventional PCR, TaqMan qPCR, and the CAPRV2023 EmOSP-RT-EmDEA system across the water, liver tissue, and feces samples. Results were coded as 1 for positive and 0 for negative. Statistical significance was determined at a significance level of 0.05, with *p*-values used to assess differences in the performance of the detection methods.

### 2.7. Clinical Samples and Ethics Statement

All fish were anesthetized using eugenol (clove oil) and humanely euthanized prior to dissection, in strict accordance with the approved animal ethics protocol. All animal experiments were conducted strictly following the recommendations in the ‘Guide for the Care and Use of Laboratory Animals’ set by the National Institutes of Health. All fish experiments were approved by the Animal Ethics Committee of Guangdong Provincial Key Laboratory of Aquatic Animal Disease Control and Healthy Culture, approval code: (20240621)006, approval date: 21 June 2024.

## 3. Results

### 3.1. Screening of RNA and EmDEA Primers

#### 3.1.1. Screening of RNA Primer

**Figure 2:**
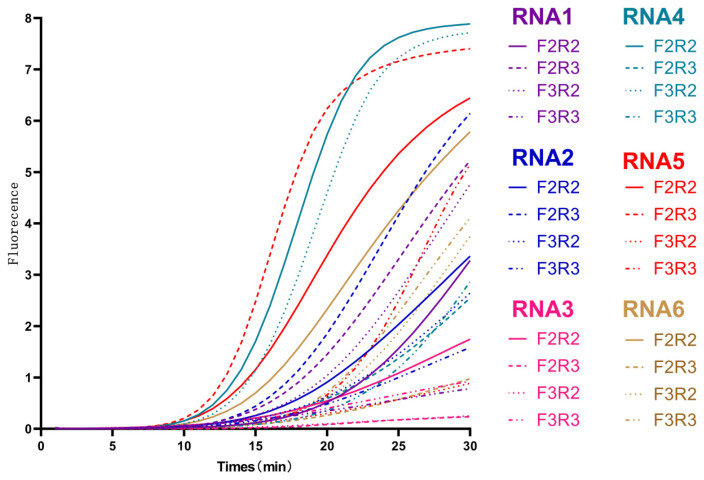
**Screening of RNA primers.** Amplification curves show reaction time on the X-axis and fluorescence intensity on the Y-axis. Each curve represents an average of three replicates.

Primer sets generating amplification signals with a Tt under 30 min were considered positive. Among these, the set with the lowest Tt was selected as the optimal primer for subsequent experiments due to its fastest amplification kinetics and highest reaction efficiency. Each combination was tested in triplicate to ensure reproducibility. Results of RNA primer screening are presented in Figure 2. Combinations involving RNA4 and RNA5 exhibited the shortest Tt and highest maximum fluorescence values among tested primers. Consequently, RNA4 and RNA5 were selected for further experiments based on these findings.

#### 3.1.2. Screening of EmDEA Primer

**Figure 3:**
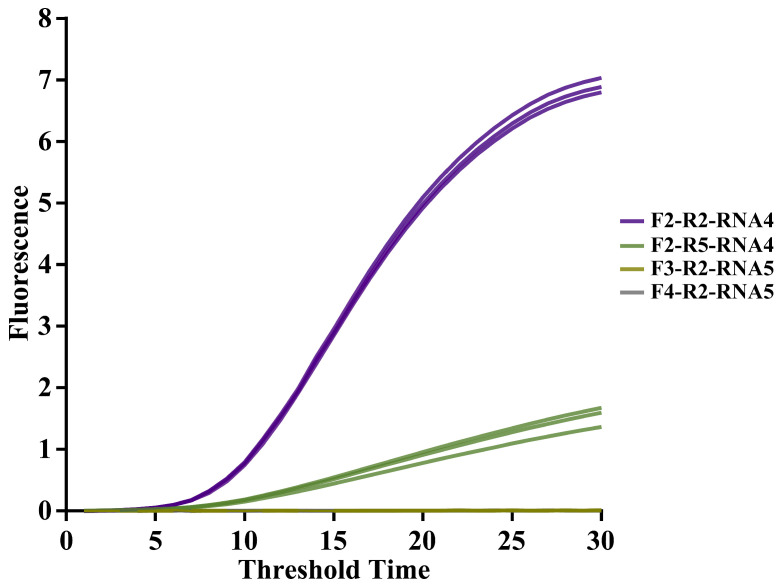
**Screening of CAPRV2023-EmDEA primer combinations.** Amplification curves show reaction time on the X-axis and fluorescence intensity on the Y-axis. Each curve represents the average of three replicates.

The primer pair with the shortest Tt identified in Section 3.1.1 was further evaluated. Primer sets producing amplification signals with Tt below 30 min were considered positive. Using low Tt as the primary screening criterion, EmDEA was conducted with various primer pairs. As shown in Supplementary Table S1, six highly sensitive primer combinations were identified for further evaluation. Using a standard plasmid with 10 copies, four primer pairs—RNA4-F2-R2, RNA4-F2-R5, RNA5-F3-R2, and RNA5-F4-R2—were selected for subsequent experiments due to their ability to amplify at low copy numbers (Figure 3).

### 3.2. CAPRV-RT-EmDEA

#### 3.2.1. RT-EmDEA Primer

**Figure 4:**
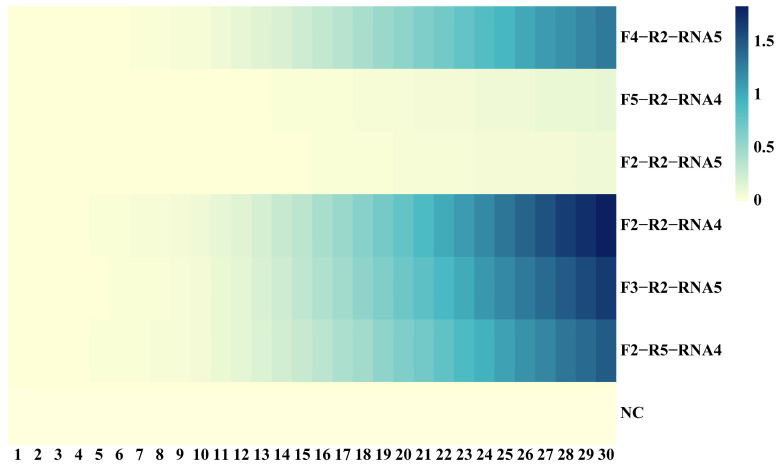
**Screening of primer combinations with high sensitivity in the EmDEA reaction combined with the reverse transcription (RT) reaction.** The heatmap illustrates the detection performance of six primer combinations at a concentration of 10 copies. Fluorescence intensity values are plotted against the corresponding threshold times, with color gradients indicating relative detection efficiency—darker colors represent stronger detection signals and shorter threshold times.

Primer combinations selected in Section 3.1 were evaluated within the RT-EmDEA system using an RNA standard sample (prepared as described in Section 2.4.1) containing 10^4^ copies. As shown in Figure 4, only the F2-R2/F2-RNA4 combination produced a clear fluorescence amplification curve. F2-R5-RNA4 showed reduced sensitivity, while the other two pairs failed to amplify. Therefore, F2-R2-RNA4 was chosen as the optimal primer combination for the CAPRV2023-RT-EmDEA detection system.

#### 3.2.2. Sensitivity of the CAPRV2023-RT-EmDEA

**Figure 5:**
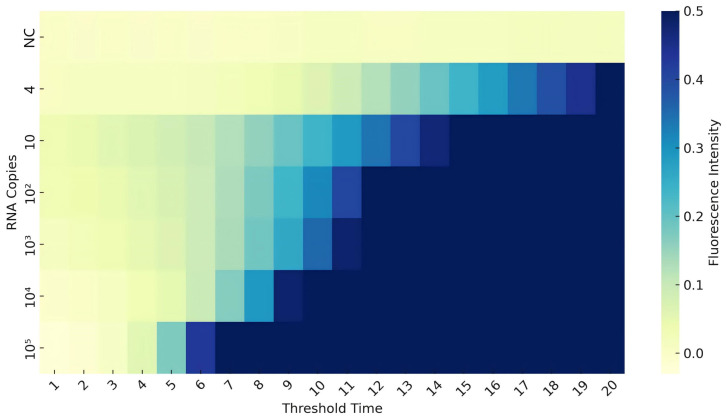
**Sensitivity threshold of the CAPRV2023-RT-EmDEA reaction.** Gradient-diluted standard plasmids were used as templates for the EmDEA reaction. The heatmap displays fluorescence values plotted against threshold time, illustrating the detection range and sensitivity. Fluorescence signals were detectable at as low as 4 copies.

The sensitivity of the CAPRV2023-RT-EmDEA system was assessed using a serially diluted RNA standard sample. As shown in Figure 5, the detection limit was determined to be 4 copies/μL of RNA template under optimized reaction conditions, establishing this as the system’s detection threshold.

### 3.3. Enzyme-Mediated One-Step Sample Processing

#### 3.3.1. Minimally Invasive Sample

**Figure 6:**
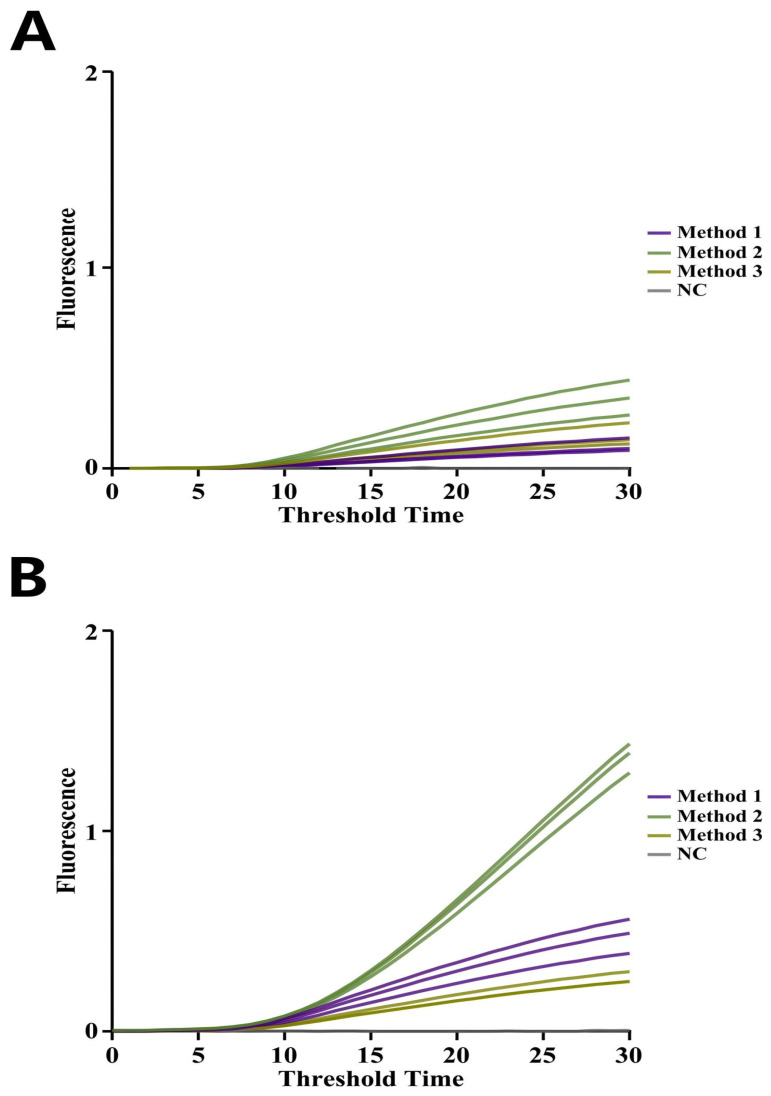
**Optimization of EmOSP for nucleic acid release from minimally invasive samples.** Threshold time was plotted on the X-axis and fluorescence intensity on the Y-axis. Each curve represents the average of three replicates. (**A**) Gill samples (10^4.83 ± 0.05^ copies/mg). (**B**) Fecal samples (10^4.76 ± 0.07^ copies/mg).

RNA copy numbers in infected fish samples were quantified by TaqMan real-time PCR, and the extraction efficiency of minimally invasive samples processed using various EmOSP methods was evaluated with the CAPRV2023-RT-EmDEA system. In gill filament samples (10^4.83 ± 0.12^ copies/mg), none of the methods yielded sufficient nucleic acids for reliable amplification (Figure 6A). Conversely, in fecal samples (10^4.76 ± 0.11^ copies/mg), Methods 1 and 3 showed comparable extraction efficiencies, with Method 2 performing best (Figure 6B).

#### 3.3.2. Invasive Sample

**Figure 7:**
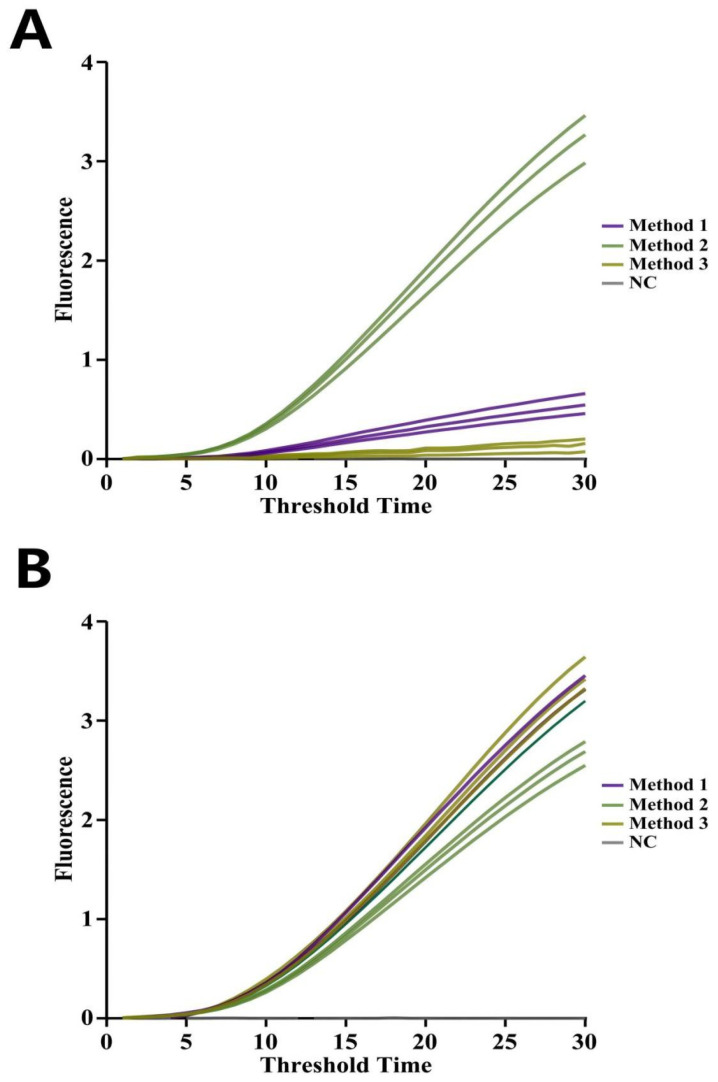
**Optimization of EmOSP for nucleic acid release from invasive samples.** Threshold time was plotted on the X-axis and fluorescence intensity on the Y-axis. Each curve represents the average of three replicates. (**A**) Liver samples (10^6.03 ± 0.16^ copies/mg). (**B**) Spleen samples (10^6.11 ± 0.08^ copies/mg).

For spleen samples (10^6.03 ± 0.16^ copies/mg), RNA was successfully extracted in quantities sufficient for downstream detection (Figure 7B). For liver samples (10^6.07 ± 0.08^ copies/mg), Methods 1 and 3 demonstrated similar efficiencies, while Method 2 resulted in significantly longer Tt, indicating lower detection efficiency (Figure 7A).

#### 3.3.3. Environmental Sample

**Figure 8:**
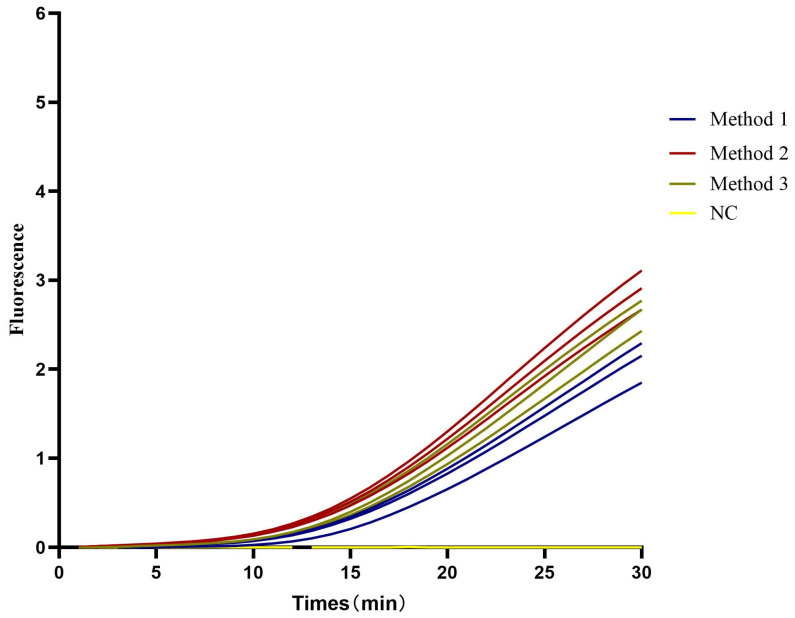
**Optimization of EmOSP for nucleic acid release from environmental samples.** Threshold time was plotted on the X-axis and fluorescence intensity on the Y-axis. Each curve represents the average of three replicates.

RNA copy numbers in filtered seawater samples from CAPRV2023 disease outbreaks were quantified by TaqMan real-time PCR, and extraction efficiency of these samples processed with different EmOSP methods was assessed by the CAPRV2023-RT-EmDEA system. The seawater sample, with 33 ppt salinity and RNA concentration of 4.2 × 10^3^ copies/μL, showed similar extraction efficiencies across methods, with Method 2 performing the best (Figure 8).

**Figure 9:**
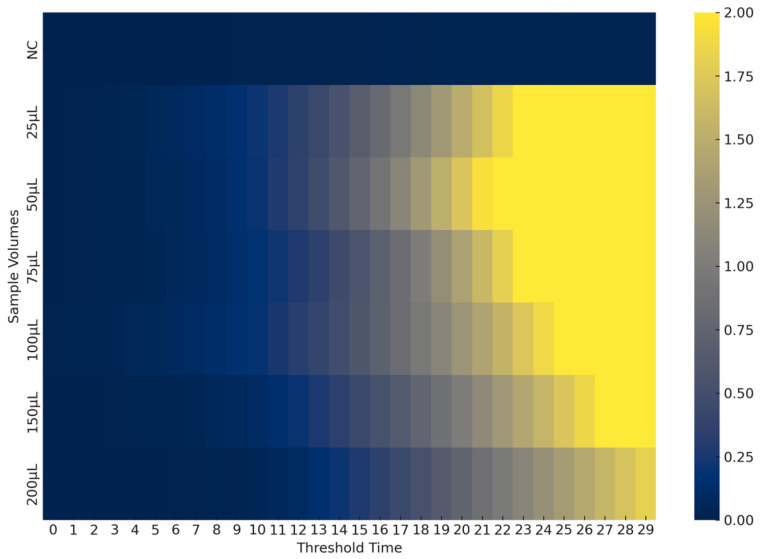
**Optimization of EmOSP treatment for environmental water samples.** The heatmap displays fluorescence intensity plotted against threshold time, providing a clear comparison. The best nucleic acid release was observed when 50 μL of EmOSP was added.

To optimize seawater sample volume for EmOSP, CAPRV2023-containing seawater was serially diluted with sterile seawater (33 ppt salinity) for detection. As shown in Figure 9, 50 μL of seawater was optimal for lysis solution testing in the CAPRV2023-RT-EmDEA system.

**Figure 10:**
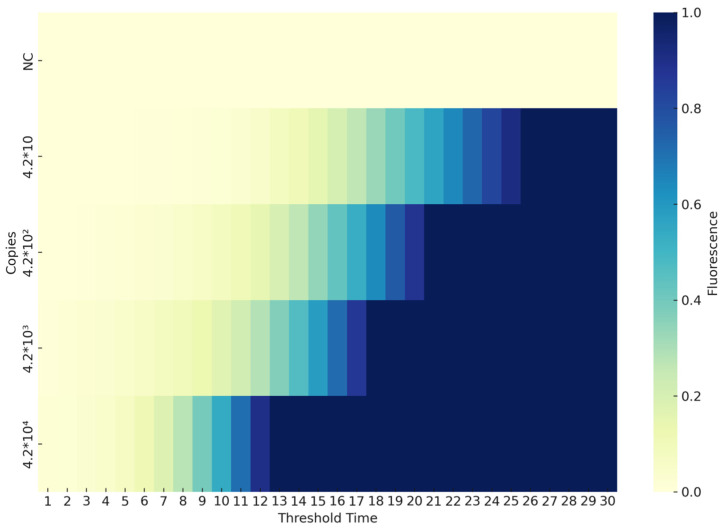
**Detection limit of the CAPRV2023-EmOSP-RT-EmDEA detection system for environmental samples.** The heatmap represents fluorescence values plotted against threshold time. Fluorescence signals were detectable at 4.2 copies.

The detection limit of the EmOSP-CAPRV2023-RT-EmDEA system was 4.2 × 10^1^ copies/μL under these optimized conditions for environmental samples (Figure 10).

### 3.4. CAPRV2023 EmOSP-RT-EmDEA Detection System

#### 3.4.1. Specificity of the CAPRV2023 EmOSP-RT-EmDEA Detection System

**Figure 11:**
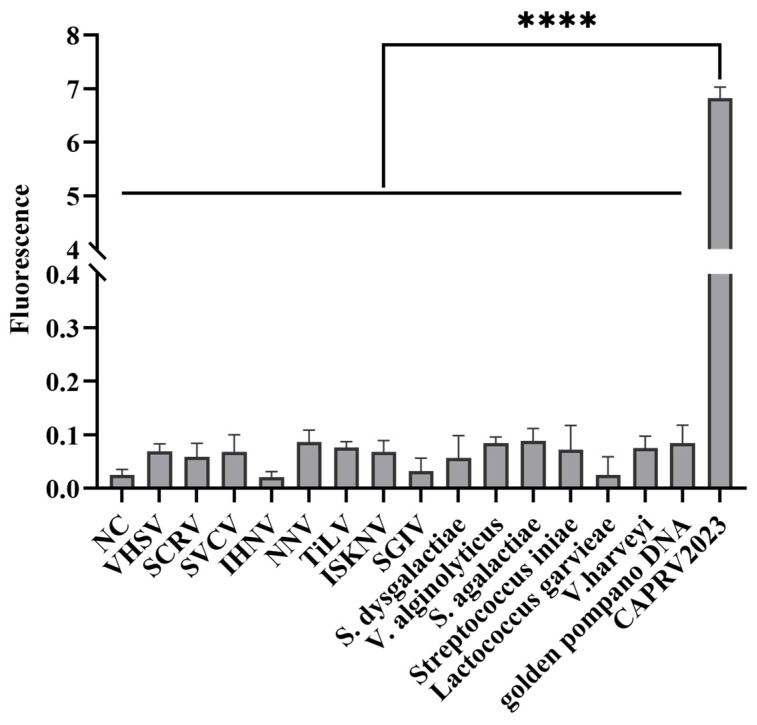
**Specificity test of the CAPRV2023-EmOSP-RT-EmDEA detection system.** Y-axis represents the fluorescence signals in the bar chart. Significant fluorescence signals were observed only for CAPRV2023, whereas no fluorescence was detected for other pathogens, confirming the high specificity of the CAPRV2023-EmOSP-RT-EmDEA detection system. Statistical significance, indicated by the asterisks (*), (****) represents a comparison between CAPRV2023 and all other pathogens, with a *p* < 0.0001.

Specificity was evaluated using a panel of common golden pompano pathogens and several viruses phylogenetically related to CAPRV2023 to ensure clinical and molecular specificity. As shown in Figure 11, amplification curves appeared exclusively for CAPRV2023; no amplification was detected for any other pathogens.

#### 3.4.2. Repeatability of the CAPRV2023 EmOSP-RT-EmDEA Detection System

**Figure 12:**
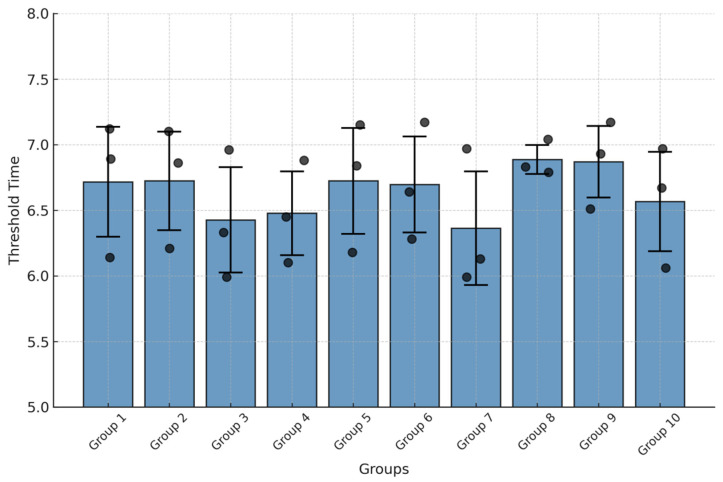
**Reproducibility test of the CAPRV2023-EmOSP-RT-EmDEA detection system.** The bar chart visualizes the results of 10 reproducibility tests, with threshold time on the Y-axis for each group. Specifically, include that the black dots represent individual data points, and the error bars indicate the standard deviation (SD).

**Figure 13:**
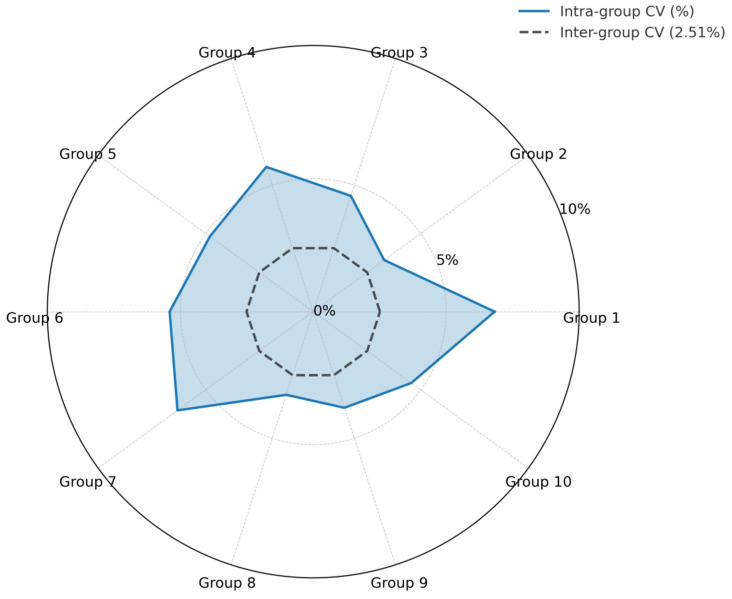
**Reproducibility visualization of the CAPRV2023-EmOSP-RT-EmDEA detection system.** Blue solid line and filled area represent intra-group coefficient of variation (CV, %); black dashed line and circle represent inter-group CV.The threshold time for each group from the reproducibility test of the CAPRV2023-EmOSP-RT-EmDEA detection system was recorded. The intra- and inter-group coefficient of variation (CV) were calculated and visualized in a radar chart.

Both intra-assay repeatability and inter-assay reproducibility of the CAPRV2023 EmOSP-RT-EmDEA detection system were evaluated by calculating the CV of Tt values, using clinical liver samples containing (10^7.59 ± 0.12^ copies/mg) of viral RNA. Ten independent reaction groups were tested, each performed in triplicate. As shown in Figure 12, the Tt values across inter- and intra-groups exhibited good consistency with minimal variation. The CVs for intra-assay ranged from 3.29% to 6.82%, with standard deviations of 0.13 to 0.58 (Figure 13). The CV for inter-assay was 2.51% (Figure 13).

#### 3.4.3. Application

**Figure 14:**
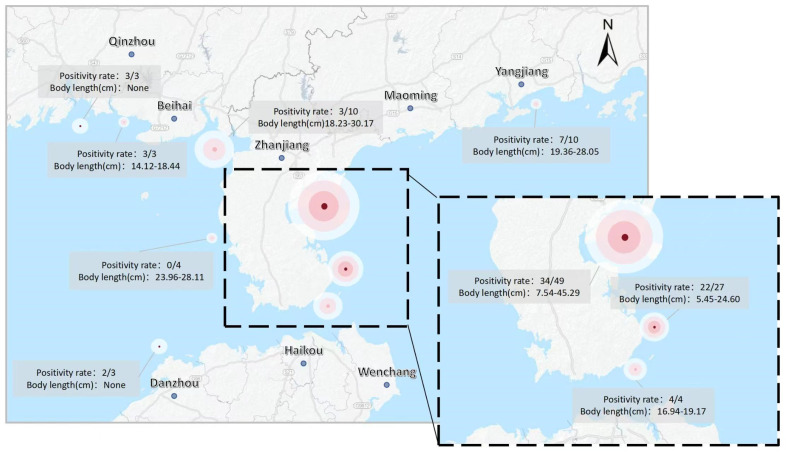
**Spatial distribution of CAPRV2023 in cage-farmed golden pompano and corresponding aquaculture water samples at different locations in the South China Sea over a complete farming cycle.**

To comprehensively assess the distribution and detectability of CAPRV2023 in golden pompano under real-world aquaculture conditions, 113 clinical and environmental samples were collected from 37 geographically distinct aquaculture sites across China. These sites spanned more than one production cycle and included golden pompano of all developmental stages (Figure 14). All samples were tested using three molecular assays: conventional PCR, TaqMan qPCR, and the EmOSP-RT-EmDEA system.

As summarized in Supplementary Table S2, the EmOSP-RT-EmDEA system demonstrated a markedly higher positive detection rate in seawater samples (76%) compared to conventional PCR (16%) and was nearly equivalent to TaqMan qPCR (79%). Statistical analysis using Fisher’s exact test confirmed no significant difference in seawater detection rates between EmOSP-RT-EmDEA and TaqMan qPCR (*p* = 0.7351), while both methods significantly outperformed conventional PCR (*p* < 0.0001). Detailed comparative data are provided in Supplementary Table S2 for clarity.

A similar trend was observed in spleen and fecal samples. The EmOSP-RT-EmDEA system achieved positive rates of 69.03% and 68.14%, respectively, consistent with TaqMan qPCR results. Fisher’s exact test revealed no significant differences between EmOSP-RT-EmDEA and TaqMan qPCR for these samples (*p* = 1.000 for both spleen and fecal samples), while both showed significantly higher sensitivity than conventional PCR (*p* = 0.0001 for spleen; *p* < 0.0001 for feces).

Collectively, these results demonstrate that the EmOSP-RT-EmDEA detection system provides field-applicable sensitivity comparable to TaqMan qPCR across environmental and clinical matrices, making it a promising alternative for rapid CAPRV2023 surveillance in aquaculture environments.

## 4. Discussion

A recent outbreak of a devastating viral disease caused by CAPRV2023 has severely impacted net cage-cultured golden pompano in China, posing a significant threat to the aquaculture industry. As reported by Sun et al. [1,2], golden pompano infected with CAPRV2023 exhibit high mortality rates within 7 to 10 days. Currently, no effective prevention or control measures exist for this epidemic. Therefore, early detection of this emerging virus during initial infection stages, coupled with timely intervention, is crucial for sustainable development of golden pompano aquaculture. In this study, a novel nucleic acid assay for CAPRV2023 based on a dual-signal amplification technique was developed and tested in the field. The assay demonstrated strong specificity, high sensitivity, excellent repeatability, and stability.

The G protein of fish rhabdoviruses is essential for membrane fusion and viral virulence and acts as a key antigen inducing virus-neutralizing antibodies and host immunity [14,15,16,17,18,19]. Given its high transcription levels in infected hosts, the G protein gene is an ideal molecular detection target, previously applied for IHNV and VHSV detection [20,21]. In this study, the CAPRV2023 EmOSP-RT-EmDEA detection system targeting the G protein gene showed high specificity and sensitivity, detecting the virus in infected fish without cross-amplification of other fish pathogens.

**Figure 15:**
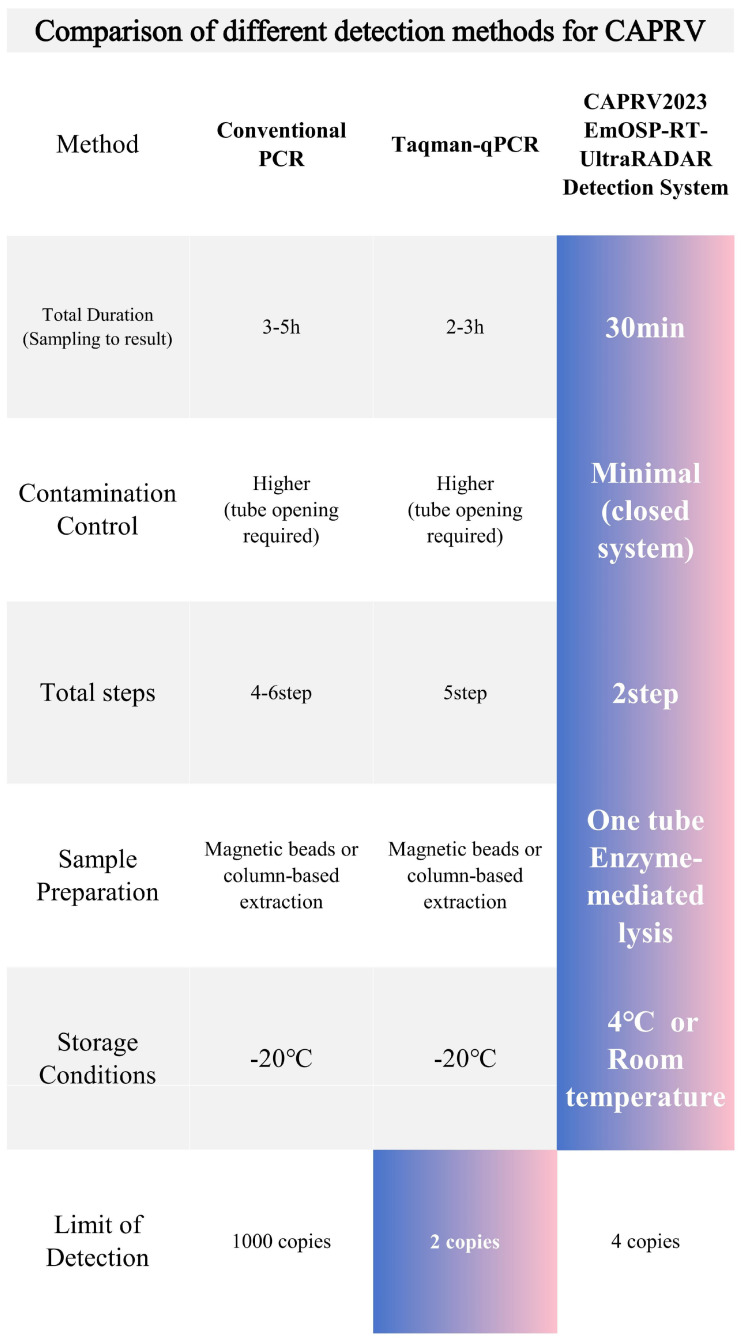
**Comparison of the advantages and disadvantages of different CAPRV detection methods.**

**Figure 16:**
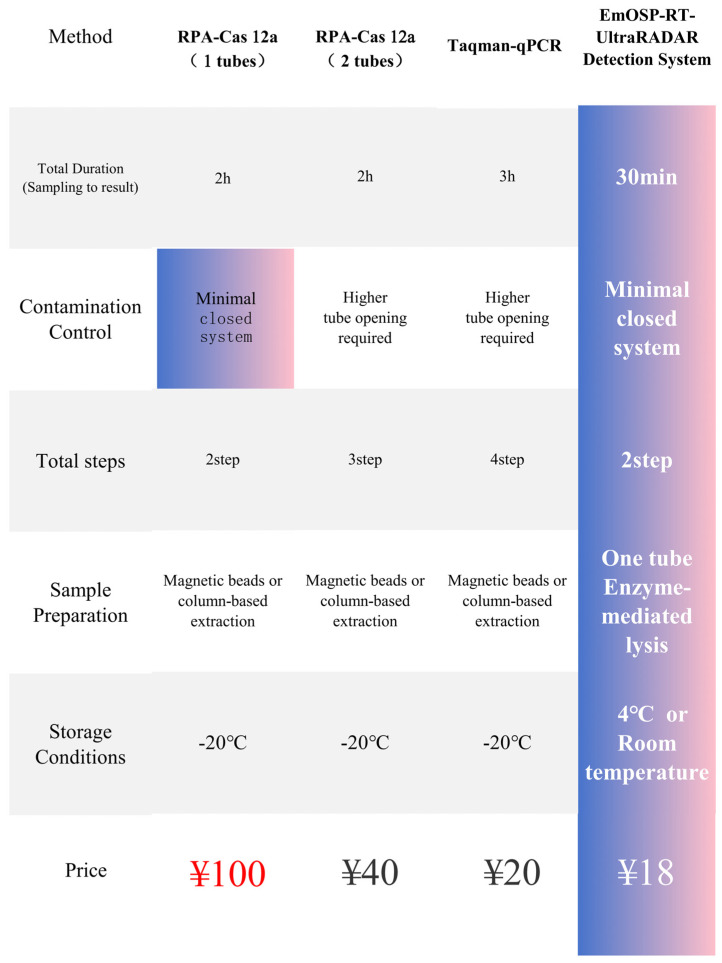
**Comparison of the advantages and disadvantages of different rapid detection methods.**

To address limitations of existing CAPRV2023 diagnostics, we evaluated conventional PCR, TaqMan qPCR, and CRISPR-Cas platforms. Conventional PCR and TaqMan qPCR are widely used, their multi-step workflows require RNA extraction, reverse transcription, and amplification in open-tube formats. These procedures increase aerosol contamination risk and involve time-consuming, instrument-dependent operations [22,23]. Dye-based qPCR detects total amplified product via fluorescence intensity, whereas probe-based qPCR uses hydrolysis probes [24]. Both rely solely on product accumulation for signal generation. Although TaqMan qPCR offers high sensitivity (2 copies/μL) and strong quantification, it requires precise thermal cycling equipment, enzyme storage at −20 °C, and at least three processing steps (extraction, reverse transcription, and amplification), limiting field applicability (Figure 15). The RPA–Cas12a detection approach can be categorized into two formats. The first is a two-tube method, in which RPA is performed first, followed by opening the tube to add the Cas12a reaction mixture. The second is a one-tube method, which eliminates the need for tube opening but requires a substantially higher concentration of Cas12a protein, resulting in a significantly increased cost (Figure 16). Detection of environmental samples using conventional methods is even more complex. For instance, TaqMan qPCR requires raw seawater to undergo filtration (0.22 μm pore size), ultrafiltration concentration, RNA extraction, reverse transcription, and amplification—five separate steps requiring specialized equipment. These additional steps increase cost, cross-contamination risk, and total turnaround time.

In contrast, RPA enables rapid isothermal nucleic acid amplification at constant low temperatures (typically around 37–42 °C) [25]. RPA eliminates the need for thermal cyclers and significantly reduces reaction time [25]. However, RPA is known to exhibit a relatively high false-positive rate due to nonspecific primer interactions under isothermal conditions, especially in complex sample matrices [26]. To improve specificity, many RPA-based methods still rely on open-tube formats for downstream detection and incorporate CRISPR-Cas enzymes for post-amplification detection [27,28,29]. These CRISPR-Cas systems enhance signal fidelity through collateral cleavage mechanisms but require multi-step workflows [27,28,29]. Reopening the reaction tube after RPA to introduce the CRISPR-Cas detection system poses a substantial contamination risk, as the reaction contains high-copy, low-molecular-weight amplicons that are highly susceptible to aerosolization and environmental dispersion [10,11,29,30,31]. Furthermore, these systems depend on temperature-sensitive reagents and specialized components, such as purified Cas proteins and guide RNAs, limiting their practicality in field settings [32,33,34].

To overcome these limitations, we developed the CAPRV2023 EmOSP-RT-EmDEA detection system, which integrates enzyme-mediated one-step sample processing, reverse transcription, and duplex exponential isothermal amplification into a fully enclosed, two-step workflow. This design eliminates the requirement for RNA purification or centrifugation, thereby minimizing contamination risk by avoiding tube reopening. It enables direct input of raw biological or environmental samples through their addition to lysis buffer tubes, where a simplified enzyme-mediated lysis step is performed. The resulting lysate can be directly used in downstream amplification without requiring conventional magnetic bead or column-based nucleic acid extraction, simplifying the workflow and reducing equipment needs (Figure 1).

As illustrated in Figure 15, the EmOSP-RT-EmDEA system delivers results in approximately 30 min with only two handling steps. It operates without thermal cyclers and supports reagent storage at 4 °C or room temperature, reducing equipment and cold-chain requirements. Compared to conventional protocols—which typically involve 3–5 discrete operations including extraction, reverse transcription, and amplification—the EmOSP approach dramatically streamlines the workflow and reduces the need for highly trained personnel. The EmOSP-RT-EmDEA system incorporates a dual-signal amplification mechanism, wherein cDNA is transcribed into RNA to exponentially enhance detection sensitivity. This strategy enables identification of low-abundance viral targets, minimizing false negatives and supporting robust field testing. The system achieved a detection limit of 4 copies/μL, closely approaching TaqMan qPCR (2 copies/μL), exceeding SYBR Green I qPCR by over 20-fold [1,2], and vastly outperforming conventional PCR (1000 copies/μL). These sensitivity advantages, along with excellent intra-assay consistency, considerably enhance the assay’s applicability for rapid, on-site diagnosis in aquaculture environments.

The assay was rigorously validated across various biological and environmental matrices. Gill tissues were found to be unsuitable for effective nucleic acid release due to low nucleic acid yield, whereas feces proved to be a minimally invasive and reliable sampling alternative. As part of a large-scale surveillance initiative, 113 clinical samples representing all life stages of golden pompano were collected from 37 aquaculture sites along the South China Sea, covering more than one complete production cycle (Figure 14). This broad geographic and temporal sampling demonstrated the robustness and field applicability of the EmOSP-RT-EmDEA system. As summarized in Supplementary Table S2, the system achieved positive detection rates of 76.0% in seawater, 69.03% in spleen tissue, and 68.14% in feces, closely matching the TaqMan qPCR results (79.0%, 69.03%, and 68.14%, respectively), and significantly outperforming conventional PCR (16.0%, 40.71%, and 32.7%, respectively). These results highlight the reliability and diagnostic effectiveness of the EmOSP-RT-EmDEA system under real-world aquaculture conditions.

In environmental testing, the EmOSP-RT-EmDEA system achieved a 76.0% positive detection rate in raw seawater samples without any pre-concentration steps. Although TaqMan qPCR showed slightly higher detection rates (79.0%), this advantage can be attributed to its use of ultrafiltration-based viral concentration prior to amplification [35]. Importantly, the EmOSP-RT-EmDEA system demonstrated the capacity to detect as few as 4.2 copies per sample directly, underscoring its sensitivity and suitability for on-site aquaculture monitoring. This capability aligns well with epidemiological needs, as prior studies have shown that CAPRV2023 infection can be initiated via waterborne exposure [1,2]. Therefore, effective detection of free virus in seawater—without complex sample processing—provides a practical early-warning approach for disease control in aquaculture operations.

In summary, the EmOSP-RT-EmDEA system offers a closed-tube, rapid, and highly sensitive nucleic acid detection platform that simplifies sample processing, minimizes contamination risk, and requires minimal equipment. These advantages establish it as an effective diagnostic and monitoring tool for CAPRV2023 in both clinical and environmental aquaculture settings. Moreover, the EmOSP-RT-EmDEA system provides a reliable and field-deployable molecular approach for monitoring viral pathogens in aquaculture and shows great potential for seafood hygiene surveillance and raw-material quality control throughout the aquatic food production chain.

## Figures and Tables

**Table 1:** **Primer sequences used in this study.**

Types of Sequence	Primer Names	Sequence
**Forward primer sequence**	CAP-ULTRA-F1	5’-CAACCTATCCCACCAAGAACACTGCTGA-3’
	CAP-ULTRA-F2	5’-ATCCCACCAAGAACACTGCTGAGGGACG-3’
	CAP-ULTRA-F3	5’-CCAAGAACACTGCTGAGGGACGAATCTT-3’
	CAP-ULTRA-F4	5’-ACACTGCTGAGGGACGAATCTTTACAAG-3’
	CAP-ULTRA-F5	5’-CTGAGGGACGAATCTTTACAAGAAAGGC-3’
	CAP-ULTRA-F6	5’-GACGAATCTTTACAAGAAAGGCAGATCA-3’
**RNA probe sequences**	2-GD-RNA1	5’6-FAM-rApp -CACAAUCAUAAAGGCGGUUGCGCCUGGA-3`BHQ1
	2-GD-RNA2	5’6-FAM-rApp -CAUAAAGGCGGUUGCGCCUGGACACCAU-3`BHQ1
	2-GD-RNA3	5’6-FAM-rApp -GGCGGUUGCGCCUGGACACCAUCCAUGG-3`BHQ1
	2-GD-RNA4	5’6-FAM-rApp -UGCGCCUGGACACCAUCCAUGGGGACUU-3`BHQ1
	2-GD-RNA5	5’6-FAM-rApp -UGGACACCAUCCAUGGGGACUUACCAAG-3`BHQ1
	2-GD-RNA6	5’6-FAM-rApp -CCAUCCAUGGGGACUUACCAAGGCCUGC-3`BHQ1
**Reverse primer sequence**	CAP-ULTRA-R1	5’-AAGCTAATACGACTCACTATAGGGTCCATTGTTCTCCAGCAAACTGCAGTGT-3’
	CAP-ULTRA-R2	5’-AAGCTAATACGACTCACTATAGGGTCCTGATCCATTGTTCTCCAGCAAACTG-3’
	CAP-ULTRA-R3	5’-AAGCTAATACGACTCACTATAGGGGGTCTGTCCTGATCCATTGTTCTCCAGC-3’
	CAP-ULTRA-R4	5’-AAGCTAATACGACTCACTATAGGGCCCCGAGGTCTGTCCTGATCCATTGTTC-3’
	CAP-ULTRA-R5	5’-AAGCTAATACGACTCACTATAGGGTGAGGTCCCCGAGGTCTGTCCTGATCCA-3’
	CAP-ULTRA-R6	5’-AAGCTAATACGACTCACTATAGGGTTTGGATGAGGTCCCCGAGGTCTGTCCT-3’
**Taq Man-qPCR primer**	CAP-GQF	5’-TGCATGATCGGTCCATGGACTG-3’
	CAR-GQR	5’-GTTTGGACTCTCTAGCTAGCTAG-3’
	CAR-GProbe	5’6-FAM-GTGGGGTACGATCGCATCCCAGATGATCGATG-3`BHQ1
**CAPRV-G-Clone**	CAP-G-COPY-F	5’-CGCAACCGCCTTTATGATTG-3’
	CAP-G-COPY-R	5’-GTCCCCTTCCTGGGTGATGAGG-3’

**Table 2:** **Reverse transcription primers used in RT-EmDEA reaction.**

Types of Primers	Primer Names	Sequence
**RT-Primer**	CAP-RT1	CCACCA
	CAP-RT2	AACTTT
	CAP-RT3	AATGAG
	CAP-RT4	CATATT
	CAP-RT5	CCCATC
	CAP-RT6	TTGGCA
	CAP-RT7	TGACCC
	CAP-RT8	TCGTCC
	CAP-RT9	TAACCA
	CAP-RT10	TGATTG
	CAP-RT11	CACAAT
	CAP-RT12	TTTCAT
	CAP-RT13	CTCCCG
	CAP-RT14	AAGGCA
	CAP-RT15	TGTGAG
	CAP-RT16	CAATCT
	CAP-RT17	TCTGAG
	CAP-RT18	GAGACT
	CAP-RT19	GGTAGG
	CAP-RT20	TGACAG

## Data Availability

The original contributions presented in the study are included in the article, further inquiries can be directed to the corresponding author.

## Supplementary Materials

The following supporting information can be downloaded at: https://www.mdpi.com/article/10.3390/foods14223929/s1, Supplementary Table S1. Screening forward and reverse primers of CAPRV2023-EmDEA reaction. (A) Reverse primer screening. (B) Forward primer screening. Supplementary Table S2. Virus Detection in clinical samples. Comparison of the results of TaqMan qPCR, conventional PCR, and CAPRV2023-EmOSP-RT-EmDEA detection system for virus detection in various clinical sample types. The viral load (Log copies/μL) for each sample type and the threshold time (Tt) for each method are presented. The positivity rates for each method are also provided, with overall detection rates summarized at the bottom.

## References

[1] Sun H., Fred B., Wang H., Huang J., Wu Z., Wu D., Lu Y., Jian J., Huang Y. (2025). One-step and two-step qPCR assays for CAPRV2023: Development and application in full-cycle epidemiological surveillance of golden pompano. Front. Vet. Sci..

[2] Sun H., Huang J., Wang H., Zhang Y., Fei Q., Zhou J., Yang L., Li Y., Jian J., Lu Y. (2025). Mass mortality associated with *Carpione rhabdovirus* in golden pompano (*Trachinotus ovatus*) in China: First report. Aquaculture.

[3] PRC, FAS China Staff (2024). 2024 China Fishery Products Report.

[4] Bovo G., Olesen N., Jørgensen P., Ahne W., Winton J. (1995). Characterization of a rhabdovirus isolated from carpione *Salmo trutta carpio* in Italy. Dis. Aquat. Org..

[5] Daher R.K., Stewart G., Boissinot M., Bergeron M.G. (2016). Recombinase Polymerase Amplification for Diagnostic Applications. Clin. Chem..

[6] Balea R., Pollak N.M., Hobson-Peters J., Macdonald J., McMillan D.J. (2023). Development and pre-clinical evaluation of a Zika virus diagnostic for low resource settings. Front. Microbiol..

[7] Zyrina N.V., Antipova V.N. (2021). Nonspecific Synthesis in the Reactions of Isothermal Nucleic Acid Amplification. Biochemistry.

[8] Chen J.S., Ma E., Harrington L.B., Da Costa M., Tian X., Palefsky J.M., Doudna J.A. (2018). CRISPR-Cas12a target binding unleashes indiscriminate single-stranded DNase activity. Science.

[9] Li S.-Y., Cheng Q.-X., Wang J.-M., Li X.-Y., Zhang Z.-L., Gao S., Cao R.-B., Zhao G.-P., Wang J. (2018). CRISPR-Cas12a-assisted nucleic acid detection. Cell Discov..

[10] Gootenberg J.S., Abudayyeh O.O., Kellner M.J., Joung J., Collins J.J., Zhang F. (2018). Multiplexed and portable nucleic acid detection platform with Cas13, Cas12a, and Csm6. Science.

[11] Sun Z., Lin K.-F., Zhao Z.-H., Wang Y., Hong X.-X., Guo J.-G., Ruan Q.-Y., Lu L.-Y., Li X., Zhang R. (2022). An automated nucleic acid detection platform using digital microfluidics with an optimized Cas12a system. Sci. China Chem..

[12] Wang B., Wang R., Wang D., Wu J., Li J., Wang J., Liu H., Wang Y. (2019). Cas12aVDet: A CRISPR/Cas12a-based platform for rapid and visual nucleic acid detection. Anal. Chem..

[13] Habib S., Azmai M.N.A., Yasin I.-S.M., Masdor N.A., Said N.A.M., Yasid N.A. (2024). Streamlined boiling lysis DNA extraction for Gram-positive aquaculture pathogen *Streptococcus agalactiae*. Arch. Microbiol..

[14] Dhandapani G., Nguyen V.G., Kim M.C., Noh J.Y., Jang S.S., Yoon S.-W., Jeong D.G., Le Huynh T.M., Le V.P., Song D. (2023). Magnetic-bead-based DNA-capture-assisted real-time polymerase chain reaction and recombinase polymerase amplification for the detection of African swine fever virus. Arch. Virol..

[15] World Organization for Animal Health (WOAH) (2016). Manual of Diagnostic Tests and Vaccines for Terrestrial Animals.

[16] Coll J.M. (1995). The glycoprotein G of rhabdoviruses. Arch. Virol..

[17] Chico V., Martinez-Lopez A., Ortega-Villaizan M., Falco A., Perez L., Coll J.M., Estepa A. (2010). Pepscan Mapping of Viral Hemorrhagic Septicemia Virus Glycoprotein G Major Lineal Determinants Implicated in Triggering Host Cell Antiviral Responses Mediated by Type I Interferon. J. Virol..

[18] Albertini A.A.V., Baquero E., Ferlin A., Gaudin Y. (2012). Molecular and Cellular Aspects of Rhabdovirus Entry. Viruses.

[19] Purcell M.K., Laing K.J., Winton J.R. (2012). Immunity to fish rhabdoviruses. Viruses.

[20] Ahmadivand S., Palić D., Weidmann M. (2021). Molecular Epidemiology of Novirhabdoviruses Emerging in Iranian Trout Farms. Viruses.

[21] Abdulgani N., Sudaryatma P.E., Berliana N.P.S. (2024). Comparison of RT-PCR and rRT-PCR Methods in Detection of Viral Hemorrhagic Septicemia Virus (VHSV) in Marine Ornamental Fish. BIO Web Conf..

[22] Chan K.G., Ang G.Y., Yu C.Y., Yean C.Y. (2021). Harnessing CRISPR-Cas to Combat COVID-19: From Diagnostics to Therapeutics. Life.

[23] Chen L., Hu M., Zhou X. (2025). Trends in developing one-pot CRISPR diagnostics strategies. Trends Biotechnol..

[24] King D.P., Reid S.M., Hutchings G.H., Grierson S.S., Wilkinson P.J., Dixon L.K., Bastos A.D., Drew T.W. (2003). Development of a TaqMan PCR assay with internal amplification control for the detection of African swine fever virus. J. Virol. Methods.

[25] Dong J., Feng W., Lin M., Chen S., Liu X., Wang X., Chen Q. (2024). Comparative Evaluation of PCR-Based, LAMP and RPA-CRISPR/Cas12a Assays for the Rapid Detection of *Diaporthe aspalathi*. Int. J. Mol. Sci..

[26] Patchsung M., Jantarug K., Pattama A., Aphicho K., Suraritdechachai S., Meesawat P., Sappakhaw K., Leelahakorn N., Ruenkam T., Wongsatit T. (2020). Clinical validation of a Cas13-based assay for the detection of SARS-CoV-2 RNA. Nat. Biomed. Eng..

[27] Li Z., Feng W., Zhu Z., Lu S., Lin M., Dong J., Wang Z., Liu F., Chen Q. (2024). Cas-OPRAD: A one-pot RPA/PCR CRISPR/Cas12 assay for on-site Phytophthora root rot detection. Front. Microbiol..

[28] Yang Y., Wang F., Xue B., Zhou X. (2023). Field-deployable assay based on CRISPR-Cas13a coupled with RT-RPA in one tube for the detection of SARS-CoV-2 in wastewater. J. Hazard. Mater..

[29] Liu Y., Liu H., Yu G., Sun W., Aizaz M., Yang G., Chen L. (2023). One-tube RPA-CRISPR Cas12a/Cas13a rapid detection of methicillin-resistant *Staphylococcus aureus*. Anal. Chim. Acta.

[30] Wu J., Mukama O., Wu W., Li Z., De Dieu Habimana J., Zhang Y., Zeng R., Nie C., Zeng L. (2020). A CRISPR/Cas12a Based Universal Lateral Flow Biosensor for the Sensitive and Specific Detection of African Swine-Fever Viruses in Whole Blood. Biosensors.

[31] Bhardwaj P., Dhangur P., Kalichamy A., Singh R. (2025). RT-RPA Assisted CRISPR/Cas12a Based One-Pot Rapid and Visual Detection of the Pan-Dengue Virus. J. Med. Virol..

[32] Myhrvold C., Freije C.A., Gootenberg J.S., Abudayyeh O.O., Metsky H.C., Durbin A.F., Kellner M.J., Tan A.L., Paul L.M., Parham L.A. (2018). Field-deployable viral diagnostics using CRISPR-Cas13. Science.

[33] Kellner M.J., Koob J.G., Gootenberg J.S., Abudayyeh O.O., Zhang F. (2019). SHERLOCK: Nucleic acid detection with CRISPR nucleases. Nat. Protoc..

[34] Broughton J.P., Deng X., Yu G., Fasching C.L., Servellita V., Singh J., Miao X., Streithorst J.A., Granados A., Sotomayor-Gonzalez A. (2020). CRISPR–Cas12-based detection of SARS-CoV-2. Nat. Biotechnol..

[35] Othman I., Helmi A., Slama I., Hamdi R., Mastouri M., Aouni M. (2023). Evaluation of three viral concentration methods for detection and quantification of SARS-CoV-2 in wastewater. J. Water Health.

