# Supplementary material for: Integrated Enzyme-Mediated One-Step Sample Processing and Duplex Amplification System for Rapid Detection of Carpione rhabdovirus in Aquaculture-Derived Food Products
Source: Foods. 2025 Nov 17;14(22):3929. doi: 10.3390/foods14223929 (PMC12652080; doi:10.3390/foods14223929)
Supplement: Supplementary file 1 [file foods-14-03929-s001.zip › Supplementary Table S1.pdf]

Table S1.A

Forward primer	Forward primer	Reverse primer	Threshold time
RNA4	F2	R1	None
		R2	6.91±0.55
		R3	9.45±0.65
		R4	10.28±0.54
		R5	7.23±0.93
		R6	None
	F3	R1	None
		R2	9.02±0.35
		R3	10.11±0.76
		R4	10.16±0.69
		R5	10.32±1.21
		R6	None
RNA5	F2	R1	None
		R2	7.64±0.51
		R3	10.73±0.48
		R4	10.63±0.93
		R5	9.47±1.37
		R6	None
	F3	R1	14.11±1.25
		R2	7.01±0.46
		F3	10.77±1.43
		F4	13.72±1.59
		F5	14.27±2.28
		F6	None

.

Table S1.B

Forward primer	Forward primer	Reverse primer	Tt for different template concentrations
RNA4	R2	F1	14.36±2.93
		F2	7.79±0.58
		F3	12.23±0.77
		F4	10.78±0.61
		F5	8.73±0.67
		F6	12.33±1.58
	R5	F1	None
		F2	8.57±0.45
		F3	10.51±1.21
		F4	11.16±1.06
		F5	13.43±2.45
		F6	None
RNA5	R2	F1	15.89±2.14
		F2	8.42±0.41
		F3	8.63±0.44
		F4	8.24±0.79
		F5	12.47±0.89
		F6	11.52±1.28
	R5	F1	None
		F2	11.01±0.72
		F3	10.15±1.74
		F4	11.53±1.63
		F5	12.57±2.25
		F6	14.11±1.46

Table S1. Screening forward and reverse primers of CAPRV2023-EmDEA reaction. (A) Reverse primer screening. (B) Forward primer screening
