# Supplementary material for: Integrated Enzyme-Mediated One-Step Sample Processing and Duplex Amplification System for Rapid Detection of Carpione rhabdovirus in Aquaculture-Derived Food Products
Source: Foods. 2025 Nov 17;14(22):3929. doi: 10.3390/foods14223929 (PMC12652080; doi:10.3390/foods14223929)
Supplement: Supplementary file 1 [file foods-14-03929-s001.zip › Supplementary Table S2.pdf]

Sample Collection Period	Region	Sample	Water Sample Virus Detection			Spleen Tissue Sample Virus Detection			Feces Sample Virus Detection			Body length(cm)
			Conventional PCR	Taqman-qPCR (Log Copies/μL)	CAPRV2023 EmOSP-RT-UltraRADAR Detection System	Conventional PCR	Taqman-qPCR (Log Copies/mg)	CAPRV2023 EmOSP-RT-UltraRADAR Detection System	Conventional PCR	Taqman-qPCR (Log Copies/mg)	CAPRV2023 EmOSP-RT-UltraRADAR Detection System	
2023.8	Central Zhanjiang, Guangdong	1-1	+	3.47	10.77	+	7.26	6.37	+	5.29	7.76	12.35
		1-2	+	3.07	11.76	+	7	7.18	+	5.58	7.39	17.54
		1-3	+	3.18	11.69	+	7.34	6.21	+	5.36	7.48	15.27
2023.8	South Zhanjiang,Guangdong	2-1	-	1.31	18.85	-	2.76	13.36	-	2.12	14.36	16.44
		2-2	-	1.68	16.21	-	2.85	12.89	-	2.05	14.89	17.11
		2-3	-	0.87	-	-	2.37	14.26	-	1.93	15.86	20.13
2023.9	South Zhanjiang,Guangdong	3-1	-	1.89	15.57	-	3.39	11.69	-	2.12	14.26	None
		3-2	-	1.76	16.82	-	3.15	10.55	-	2.35	13.64	None
		3-3	-	-	-	-	-	-	-	-	-	None
2023.10	Central Zhanjiang, Guangdong	4-1	+	3.31	10.86	+	7.37	7.12	+	5.26	8.46	21.65
		4-2	-	2.87	12.58	+	8	5.61	+	6.07	6.63	24.28
		4-3	-	2.68	12.13	+	7.78	5.86	+	5.89	7.32	26.77
2023.11	South Zhanjiang,Guangdong	5-1	-	-	-	-	-	-	-	-	-	None
		5-2	-	1.62	18.35	-	2.94	12.26	-	2.11	14.77	None
		5-3	-	-	-	-	-	-	-	-	-	None
2023.11	Central Zhanjiang, Guangdong	6-1	-	2.19	15.15	+	5.34	9.31	+	3.4	11.74	17.31
		6-2	-	2.43	14.23	+	5.86	8.54	+	4.03	9.93	21.1
		6-3	-	2.35	13.76	+	5.56	8.65	+	3.62	10.61	23.85
2024.4	Central Zhanjiang, Guangdong	7-1	-	1.98	16.58	+	4.25	9.22	-	2.78	11.77	None
		7-2	-	1.56	19.79	+	4.47	8.97	-	2.47	12.62	None
		7-3	-	1.9	16.22	+	5.28	8.23	+	3.28	10.27	None
2024.4	South Zhanjiang,Guangdong	8-1	-	1.16	-	-	2.84	13.57	-	1.87	16.42	9.32
		8-2	-	0.84	-	-	-	-	+	-	-	5.45
		8-3	-	-	-	-	-	-	+	-	-	8.33
2024.6	Central Zhanjiang, Guangdong	9-1	+	2.28	10.62	+	6.26	6.5	+	3.39	9.52	17.63
		9-2	+	1.79	14.42	+	6.23	7.47	+	3.14	8.27	12.76
		9-3	+	1.57	15.8	+	6.34	8.41	+	3.13	10.13	15.41
2024.6	Beihai,Guangxi	10-1	-	0.62	17.82	-	1.61	13.72	-	1.91	16.69	18.44
		10-2	-	0.68	18.1	-	1.78	12.92	-	1.95	14.31	17.63
		10-3	-	0.74	15.17	-	1.93	12.6	-	1.21	16.57	14.12
2024.6	South Zhanjiang,Guangdong	11-1	-	0.67	18	-	2.15	12.42	-	1.46	16.26	19.32
		11-2	-	0.76	17.65	-	1.89	15.84	-	1.13	16.13	16.44
		11-3	-	0.89	15.98	-	1.62	14.69	-	2.29	12.91	18.1
2024.7	South Zhanjiang,Guangdong	12-1	+	2.47	13.76	+	6.37	7.65	+	2.37	12.13	15.47
		12-2	+	2.26	11.56	+	7.23	6.98	-	2.52	10.12	16.38
		12-3	+	2.78	13.68	+	6.78	8.75	+	3.57	9.19	15.84
2024.7	Yangjiang,Guangdong	13-1	None	None	None	-	-	-	-	-	-	None
		13-2	None	None	None	-	-	-	-	-	-	None
		13-3	None	None	None	-	-	-	-	-	-	None
2024.7	South Zhanjiang,Guangdong	14-1	-	1.19	12.41	+	4.34	12.32	-	2.7	10.34	14.32
		14-2	-	1.43	14.37	+	4.86	9.03	+	3.48	8.41	15.57
		14-3	-	1.35	13.76	+	4.56	8.97	-	2.73	13.98	14.48
2024.7	Qinzhou，Guangxi	15-1	None	None	None	+	3.68	11.45	-	2.42	13.64	None
		15-2	None	None	None	+	3.47	9.41	+	3.79	12.48	None
		15-3	None	None	None	+	4.28	10.79	+	3.6	10.01	None
2024.7	Central Zhanjiang,Guangdong	16-1	+	2.47	13.83	+	6.43	7.93	-	2.39	10.81	20.23
		16-2	+	2.65	12.61	+	6.79	8.62	+	3.53	8.39	17.81
		16-3	+	2.18	13.7	+	5.87	7.62	+	3.17	12.03	19.36
2024.8	South Zhanjiang,Guangdong	17-1	-	1.44	14.52	+	4.82	11.38	+	2.97	13.85	18.7
		17-2	+	1.81	14.35	+	5.53	8.25	+	3.01	10.05	16.37
		17-3	-	1.38	15.43	+	6.03	7.03	+	3.53	11.38	19.88
2024.8	Yangjiang,Guangdong	18-1	-	1.79	13.88	+	4.85	11.78	+	3.33	9.25	19.36
		18-2	-	1.93	14.52	+	4.74	11.37	+	3.01	10.13	20.73
		18-3	-	2.1	13.54	+	4.98	9.64	+	3.26	8.39	21.65
2024.8	Hainan province	19-1	None	None	None	-	1.3	15.22	-	1.46	15.06	None
		19-2	None	None	None	-	0.85	17.21	-	-	-	None
		19-3	None	None	None	-	-	-	-	-	-	None
2024.9	South Zhanjiang,Guangdong	20-1	-	1.56	13.73	+	6.45	7.85	+	3.24	10.4	22.13
		20-2	+	2.36	12.32	+	6.73	7.67	+	3.62	12.13	23.74
		20-3	+	2.13	12.25	+	5.98	7.04	+	3.18	11.01	24.6
2024.9	Yangjiang,Guangdong	21-1	None	None	None	+	2.49	11.25	-	1.68	13.59	27.64
		21-2	None	None	None	+	2.35	13.99	-	2.11	11.07	28.05
2024.9	Central Zhanjiang,Guangdon	22-1	-	-	-	-	-	-	-	-	-	25.23
		22-2	-	-	-	-	-	-	-	-	-	26.44

	g	22-3	-	-	-	-	-	-	-	-	-	25.58
		22-4	-	-	-	-	-	-	-	-	-	24.26
2024.9	Central Zhanjiang,Guangdong	23-1	-	1.06	14.55	+	4.37	8.36	+	3.03	12.9	27.96
		23-2	-	1.64	13.2	+	5.65	6.28	+	3.36	12.74	29.02
		23-3	-	1.55	13.99	+	4.66	10.12	+	3.19	11.52	27.55
		23-4	-	1.11	14.19	+	5.27	8.31	+	3.08	12.05	28.4
2024.10	Central Zhanjiang,Guangdong	24-1	-	-	-	-	-	-	-	-	-	23.31
		24-2	-	-	-	-	-	-	-	-	-	24.44
		24-3	-	1.47	13.19	-	-	-	-	-	-	26.5
		24-4	-	-	-	-	-	-	-	-	-	28.07
		24-5	-	1.87	14.71	+	2.88	10.84	-	1.64	14.08	24.29
		24-6	-	1.75	14.74	+	3.05	11.15	+	2.87	14	23.74
2024.10	North Zhanjiang,Guangdong	25-1	-	1.26	12.86	-	1.95	15.82	-	2.49	13.9	29.55
		25-2	-	1.17	12.07	-	2.16	11.76	-	2.03	13.48	30.17
		25-3	-	-	-	-	-	-	-	-	-	29.38
		25-4	-	-	-	-	-	-	-	-	-	27.94
2024.10	Central Zhanjiang,Guangdong	26-1	-	1.33	15.81	-	1.13	14.1	-	1.29	15.69	28.6
		26-2	-	0.96	17.48	-	1.69	15.99	-	2.45	10.06	24.76
2024.10	Central Zhanjiang,Guangdong	27-1	-	1.72	13.21	-	-	-	-	-	-	25.3
		27-2	-	1.23	14.52	-	-	-	-	-	-	9.12
		27-3	-	1.38	15.83	-	-	-	-	-	-	22.54
		27-4	-	1.25	14.18	-	-	-	-	-	-	25.78
2024.11	Central Zhanjiang,Guangdong	28-1	-	1.51	14.64	-	2.54	13.18	-	1.54	13.4	29.45
		28-2	-	1.15	13.61	-	2.82	11.45	-	1.03	16.96	27.89
2024.11	West Zhanjiang,Guangdong	29-1	-	-	-	-	-	-	-	-	-	23.96
		29-2	-	-	-	-	-	-	-	-	-	24.65
		29-3	-	-	-	-	-	-	-	-	-	28.11
		29-4	-	-	-	-	-	-	-	-	-	26.38
2024.11	Central Zhanjiang,Guangdong	30-1	-	1.23	15.62	-	2.78	11.66	-	2.46	13.24	30.05
		30-2	-	1.41	14.06	-	2.22	10.17	-	1.72	14.91	29.34
		30-3	-	1.37	12.95	-	2.69	12.32	-	2.12	12.33	27.03
2024.11	North Zhanjiang,Guangdong	31-1	-	-	-	-	-	-	-	-	-	21.14
		31-2	-	0.92	16.52	-	1.37	12.49	-	1.52	13.83	22.46
		31-3	-	-	-	-	-	-	-	-	-	18.23
2024.12	Central Zhanjiang,Guangdong	32-1	-	-	-	-	-	-	-	-	-	45.29
		32-2	-	1.08	15.06	-	-	-	-	-	-	40.77
		32-3	-	0.76	19.88	-	-	-	-	-	-	41.58
2024.12	North Zhanjiang,Guangdong	33-1	-	-	-	-	-	-	-	-	-	23.69
		33-2	-	0.79	18.74	-	-	-	-	-	-	24.23
		33-3	-	0.82	17.55	-	-	-	-	-	-	26.67
2024.12	South Zhanjiang,Guangdong	34-1	-	1.48	15.92	-	4.03	12.56	-	2.5	10.24	17.67
		34-2	+	2.16	11.81	-	4.57	9.47	+	3.09	8.43	16.94
2024.12	Central Zhanjiang,Guangdong	35-1	-	0.67	16.02	-	1.26	12.52	-	1.07	14.23	8.23
		35-2	-	1.19	15.48	-	1.79	15.01	-	2.49	11.39	7.54
		35-3	-	0.86	16.69	-	1.57	14.23	-	1.56	15.4	9.76
2024.12	South Zhanjiang,Guangdong	36-1	-	1.76	15.85	-	3.02	8.69	+	3.09	9.44	18.23
		36-2	-	1.41	12.43	-	3.81	10.54	-	2.66	11.93	19.17
2025.1	Yangjiang,Guangdong	37-1	None	None	None	+	5.1	6.49	-	2.46	13.53	None
		37-2	None	None	None	+	5.31	7.53	+	2.83	13.29	None
Positive rate			16%	79.00%	76.00%	40.71%	69.03%	69.03%	34.21%	68.14%	68.14%	

Supplementary Table S2. Virus Detection in clinical samples. Comparison of the results of TaqMan qPCR, conventional PCR, and CAPRV2023-EmOSP-RT-EmDEA detection system for virus detection in various clinical sample types. The viral load (Log copies/μL) for each sample type and the threshold time (Tt) for each method are presented. The positivity rates for each method are also provided, with overall detection rates summarized at the bottom.
